# The Application of Artificial Intelligence in the Diagnosis and Drug Resistance Prediction of Pulmonary Tuberculosis

**DOI:** 10.3389/fmed.2022.935080

**Published:** 2022-07-28

**Authors:** Shufan Liang, Jiechao Ma, Gang Wang, Jun Shao, Jingwei Li, Hui Deng, Chengdi Wang, Weimin Li

**Affiliations:** ^1^Department of Respiratory and Critical Care Medicine, Med-X Center for Manufacturing, Frontiers Science Center for Disease-Related Molecular Network, West China School of Medicine, West China Hospital, Sichuan University, Chengdu, China; ^2^Precision Medicine Key Laboratory of Sichuan Province, Precision Medicine Research Center, West China Hospital, Sichuan University, Chengdu, China; ^3^AI Lab, Deepwise Healthcare, Beijing, China

**Keywords:** pulmonary tuberculosis, artificial intelligence, deep learning, radiomics, machine learning

## Abstract

With the increasing incidence and mortality of pulmonary tuberculosis, in addition to tough and controversial disease management, time-wasting and resource-limited conventional approaches to the diagnosis and differential diagnosis of tuberculosis are still awkward issues, especially in countries with high tuberculosis burden and backwardness. In the meantime, the climbing proportion of drug-resistant tuberculosis poses a significant hazard to public health. Thus, auxiliary diagnostic tools with higher efficiency and accuracy are urgently required. Artificial intelligence (AI), which is not new but has recently grown in popularity, provides researchers with opportunities and technical underpinnings to develop novel, precise, rapid, and automated implements for pulmonary tuberculosis care, including but not limited to tuberculosis detection. In this review, we aimed to introduce representative AI methods, focusing on deep learning and radiomics, followed by definite descriptions of the state-of-the-art AI models developed using medical images and genetic data to detect pulmonary tuberculosis, distinguish the infection from other pulmonary diseases, and identify drug resistance of tuberculosis, with the purpose of assisting physicians in deciding the appropriate therapeutic schedule in the early stage of the disease. We also enumerated the challenges in maximizing the impact of AI in this field such as generalization and clinical utility of the deep learning models.

## Introduction

Among the infectious diseases, tuberculosis (TB) is one of the major causes of mortality worldwide, leading to approximately 1.4 million deaths and 10 million new cases annually, according to the World Health Organization (WHO) Global Tuberculosis Report 2021 (1). In addition to the threat to public health posed by TB, the incidence of drug-resistant tuberculosis (DR-TB) continues to increase, resulting in difficulty in controlling the epidemic (2). Accurate detection methods based on bacteria, such as acid-fast bacilli or bacterial cultures, are time-consuming and condition-limited. Gene testing to identify infection or drug resistance of the pathogen-*Mycobacterium tuberculosis* (*M. tuberculosis*) is inconvenient and restricted by the laboratory environment. Although medical images, such as chest radiographs [also called chest X-ray (CXR)] and computed tomography (CT), are comparatively inexpensive and more available, in certain developing countries or backward areas, there may be no advanced medical equipment or a lack of experienced radiologists to interpret the images, and the growing medical image data may add workload to the physicians. Therefore, automated, precise, efficient, and cost-effective assistance tools devoted to TB management demand prompt exploitation.

Over the past decades, with the vigorous development of computer technology, artificial intelligence (AI) has aroused a whopping level of attention in many fields, especially in image recognition. AI systems based on medical images or other meaningful clinical information have been utilized to screen, diagnose, assess severity, and predict prognosis in multiple diseases, such as brain tumor (3, 4), pneumonia (5), lung cancer (6), cardiovascular disease (7), and even tumor metastasis (8).

In addition, for better implementation of AI in the medical field, particular ethical concerns should also be considered. With the widespread development and utilization of AI, privacy and security during the management and transmission of data, as well as the informed consent of patients are emerging as critical ethical issues. Moreover, specific psychological and legal considerations have also been proposed. For instance, when an error by the automated system leads to a false diagnosis or improper therapeutic schedule resulting in harmful consequences, this may cause a dispute over who should be responsible for that mishap. In medical practice, owing to the opaqueness of the prediction generated by the algorithm, physicians may distrust the model (9). Furthermore, to verify the clinical relevance of the models, clinical trials are required, wherein more intractable issues, such as obtaining informed consent, are present; however, only a few clinical trials involving the use of AI systems have been performed. Collectively, to guide the appropriate adoption of AI systems, the establishment of effective ethical and legal frameworks is of great urgency (10, 11).

We searched the literature in PubMed, Embase, and Web of Science using a retrieval search strategy with the following keywords: “tuberculosis” and “artificial intelligence” or “deep learning” or “radiomics” or “machine learning,” selecting quantified studies by the abstracts, and the flow diagram is demonstrated in Supplementary Figure 1. In this review, we mainly focused on approaches based on AI using CXR, CT, positron emission tomography (PET)/CT images, and genetic data associated with TB care. By describing the latest typical AI studies focusing on TB, we aimed to inform physicians and radiologists interested in AI for the precise diagnosis of TB to carry out optimal therapeutic regimens.

**FIGURE 1:**
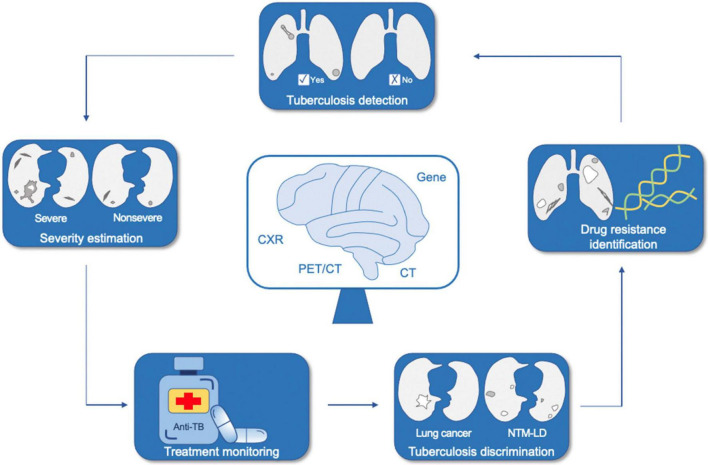
Application of artificial intelligence in tuberculosis. NTM-LD, non-tuberculous mycobacterium lung disease; CXR, chest X-ray; CT, computed tomography; PET/CT, positron emission tomography/computed tomography.

We started by briefly introducing AI, with deep learning and radiomics stressed; later, we provided a few definite examples of the application of AI in the medical field, especially in respiratory system. We then narrated the up-and-coming AI techniques in TB from three aspects according to the proposed use, namely, TB detection, discrimination between TB and other pulmonary diseases, and recognition of drug resistance of TB (Figure 1). Finally, we summarized the significance of previous studies, challenges, and prospects of developing more practical and accurate AI tools for TB in the future.

## Artificial Intelligence in a Nutshell

**FIGURE 2:**
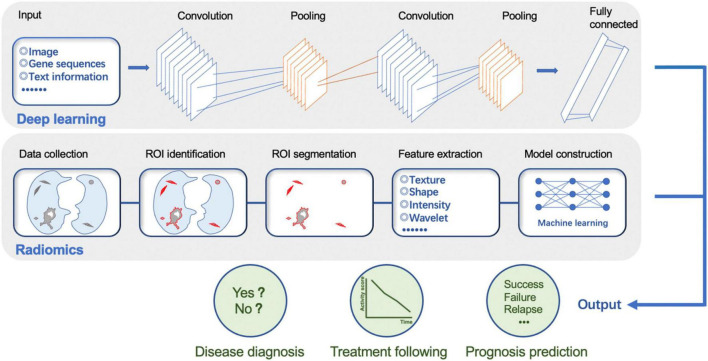
The workflow of deep learning and radiomics. ROI, region of interest.

AI is a technical science that studies and develops the theory, method, technology, and application of systems used to simulate and extend human intelligence. Deep learning, a hot topic in this field, which has been probed extensively, mostly leverages convolutional neural networks (CNNs) comprised of multiple layers, including input, convolutional, pooling, fully connected, and output layers, through which the specific predictions could derive from primary digitalized inputs, such as images, speech, gene sequences, and clinical text information (12, 13). What’s more, other plentiful sorts of machine learning algorithms, such as logistic regression (LR), random forest (RF), support vector machine (SVM), and decision tree (DT), are valuable components of AI as well (14–18). Radiomics, designed to mine pathophysiological information from medical images, includes a common process involving data collection; identification of the region of interest (ROI); ROI segmentation; feature extraction, selection, and quantification; model establishment; and prediction making in the end (19, 20). The workflow of deep learning and radiomics is displayed in Figure 2.

The prosperity of AI applied to the medical field, especially in respiratory system, has attracted substantial attention with promising results, such as detection of pulmonary nodules (21) and prediction of treatment response or outcome of lung cancer (22, 23). Meanwhile, we have made excellent achievements, including diagnosis and discrimination of 2019 novel coronavirus pneumonia (24), predetermination of epidermal growth factor receptor (EGFR) gene mutation status, programmed death ligand-1 (PD-L1) expression level, and target therapy effect in patients with lung cancer (25–27).

As a noticeable disease in this system, AI applied to TB is presented as follows and summarized briefly in Table 1.

**TABLE 1:** A brief summary of the included studies.

Section	Study proportion	Purpose	Reference standard	Primary materials	Algorithm	Evaluation indicators	References
Tuberculosis detection	48.5%	Diagnose pulmonary tuberculosis or disease evaluation	Pathogenic detection, radiology reports, clinical records, etc.	CXR and CT images	CNN and ML	AUC, sensitivity, specificity, accuracy, etc.	(31–41, 43–47)
Tuberculosis discrimination	18.2%	Discriminate between pulmonary tuberculosis and lung cancer or NTM-LD	Pathogenic detection, pathology, or follow-up confirmation	CT and PET/CT images	CNN and radiomics		(52–55, 59, 60)
Tuberculosis drug resistance prediction	33.3%	Recognize MDR-TB or drug resistance of *Mycobacterium tuberculosis* up to 14 anti-tuberculosis drugs	Drug susceptibility testing	CXR, CT images, and gene sequences	ANN, CNN, GNN, and ML		(63–65, 68–73)

*CXR, chest X-ray; CT, computed tomography; CNN, convolutional neural network; ML, machine learning; AUC, area under the curve; NTM-LD, non-tuberculous mycobacterium lung disease; PET/CT, positron emission tomography/computed tomography; MDR-TB, multi-drug resistant tuberculosis; ANN, artificial neural network; GNN, graph neural network.*

## Application of Artificial Intelligence in Pulmonary Tuberculosis

### Detection of Pulmonary Tuberculosis

Since the majority of patients with pulmonary tuberculosis (PTB) have abnormal chest CXR findings, such as cavities, centrilobular nodules, and consolidations (28), which are suggestive of the diagnosis of PTB, and CXR is comparatively widely available, WHO has recommended TB screening in high-risk populations by chest radiographs (29). Similarly, CT images demonstrate abnormalities when PTB occurs. These representative medical images are commonly utilized to train a deep learning model to detect PTB suffering. As early as 1999, an artificial neural network was exploited to predict active TB, taking advantage of radiographic findings, symptoms, and demographic variables, showing a favorable performance, which gave researchers powerful afflatus (30). Thereafter, abundant studies have been conducted to recognize the contagious disease using radiological images in slightly different forms (Table 2).

**TABLE 2:** Summary of AI applications in TB detection.

No.	References	Method	Reference standard	Dataset	Study population	Training/Validation/test cohort	Model names	Algorithm	Results
1	Lakhani and Sundaram (31)	Retrospective multi-center on CXR images	Sputum, radiology reports, radiologists, and clinical records.	1,007 participants	United States, China, and Belarus	Training: 685 Validation: 172 Test: 150	NA	CNN	AUC 0.99, Sen 97.3%, Spe 94.7%, Acc 96.0% of the ensemble method
2	Hwang et al. (32)	Retrospective multi-center on CXR images	Culture or PCR	62,433 CXR images	Korea, China, United States, etc.	Training: 60,089 Tuning: 450 Internal validation: 450 External validation: 1,444	DLAD	CNN	AUC 0.977–1.000 for TB classification, AUAFROC 0.973–1.000 for lesion localization; Sen 0.943–1.000, Spe 0.911–1.000 at high sensitivity cutoff
3	Nijiati et al. (33)	Retrospective single-center on CXR images	Symptoms, laboratory and radiological examinations	9,628 CXR images	China	Training: 7,703 Test: 1,925	NA	CNN	AUC 0.9902–0.9944, Sen 93.2–95.5%, Spe 95.78–98.05%, Acc 94.96–96.73% in the test set
4	Lee et al. (34)	Retrospective single-center on CXR images	Smear microscopy, culture, PCR, and radiologists	19,686 participants	Korea	Test: 19,686	DLAD	CNN	AUC 0.999, Sen 1.000, Spe 0.959–0.997, Acc 0.96–0.997
5	Heo et al. (35)	Retrospective single-center on CXR images	Radiologists	39,677 participants	Korea	Training: 2,000 Test: 37,677	D-CNN and I-CNN	CNN	AUC 0.9213, Sen 0.815, Spe 0.962 of D-CNN
6	Nafisah and Muhammad (36)	Retrospective multi-center on CXR images	NA	1,098 CXR images	United States, China, and Belarus	5-fold cross validation	NA	CNN	AUC 0.999, Acc 98.7%, recall 98.3%, precision 98.3%, Spe 99.0%
7	Pasa et al. (37)	Retrospective multi-center on CXR images	NA	1,104 participants	United States, China, and Belarus	5-fold cross validation	NA	CNN	AUC 0.925, Acc 86.2%
8	Rajaraman et al. (38)	Retrospective multi-center on CXR images	Radiologists	76,031 CXR images	United States and Spain	Training: test 9:1	NA	CNN	AUC 0.9274–0.9491, recall 0.7736–0.8113, precision 0.9524–0.9773, Acc 0.8585–0.8962
9	Rajpurkar et al. (39)	Retrospective multi-center on CXR images	Culture or Xpert MTB/RIF	677 participants	South Africa	Training: 563 Test: 114	CheXaid	Deep learning	AUC 0.83, Sen 0.67, Spe 0.87, Acc 0.78
10	Lee et al. (40)	Retrospective multi-center on CXR images	Sputum microscopy, culture or PCR	6,964 participants	Korea	Training: validation 7:3 Test: 455	NA	CNN	AUC 0.82–0.84, Spe 26–48.5% at the cutoff of 95% Sen in the test set
11	Yan et al. (41)	Retrospective multi-center on CT images	Culture	1,248 CT images	China and United States	Training: validation 8:2 External test: 356	NA	CNN	Acc 95.35–98.25%, recall 94.87–100%, precision 94.87–98.70%
12	Khan et al. (43)	Prospective single-center on CXR images	Culture	2,198 participants	Pakistan	Test: 2,198	qXR and CAD4TB	CNN	AUC 0.92, Sen 0.93, Spe 0.75 for qXR; AUC 0.87, Sen 0.93, Spe 0.69 for CAD4TB
13	Qin et al. (44)	Retrospective multi-center on CXR images	Xpert MTB/RIF	1,196 participants	Nepal and Cameroon	Test: 1,196	qXR, CAD4TB, and Lunit INSIGHT CXR	CNN	AUC 0.92–0.94, Sen 0.87–0.91, Spe 0.84–0.89, Acc 0.85–0.89
14	Qin et al. (45)	Retrospective multi-center on CXR images	Xpert MTB/RIF	23,954 participants	Bangladesh	Test: 23,954	qXR, CAD4TB, InferRead DR, etc.	CNN	AUC 84.89–90.81%, Sen 90.0–90.3%, Spe 61.1–74.3% when fixed at 90% Sen
15	Codlin et al. (46)	Retrospective multi-center on CXR images	Xpert MTB/RIF	1,032 participants	Viet Nam	Test: 1,032	qXR, CAD4TB, Genki, etc.	CNN	AUC 0.50–0.82, Spe 6.3–48.7%, Acc 17.8–54.7% when fixed at 95.5% Sen
16	Melendez et al. (47)	Retrospective single-center on CXR images	Culture	392 patients	South Africa	10-fold cross validation	CAD4TB	Machine learning	AUC 0.72–0.84, Spe 24–49%, NPV 95–98% when fixed at 95% Sen

*AI, artificial intelligence; TB, tuberculosis; CXR, chest X-ray; NA, not available; CNN, convolutional neural network; AUC, area under the curve; Sen, sensitivity; Spe, specificity; Acc, accuracy; PCR, polymerase chain reaction; AUAFROC, area under the alternative free-response receiver-operating characteristic curve; CT, computed tomography.*

#### Detection of Pulmonary Tuberculosis Alone

Lakhani and Sundaram (31) adopted two deep CNNs to detect PTB on CXR images. Finally, the area under the curve (AUC) achieved a significant level at 0.99 [95% confidence interval (CI) 0.96–1.00] on account of a method named “resemble,” which indicated that the ultimate PTB probability score was obtained from the two CNNs, with a different weighting of their outputs and choosing the best match. In addition, this study revealed that networks pretrained by daily color images outperformed untrained ones [AUC 0.98 pretrained vs. 0.90 untrained of AlexNet and 0.98 pretrained vs. 0.88 untrained of GoogLeNet (*P* < 0.001) in the test dataset]. Similarly, Hwang et al. (32) developed an automatic detection algorithm to classify active PTB using chest radiographs from a massive dataset containing 60,989 images which eventually manifested high performance both in lesion localization [area under the alternative free-response receiver operating characteristic curves (AUAFROC) 0.973–1.000] and disease classification (AUC 0.977–1.000), while the observer performance test showed that the algorithm had better behavior than physicians with different degrees of experience (AUC 0.993 vs. 0.664–0.925 in localization and 0.993 vs. 0.746–0.971 in classification). Another study developed an algorithm based on ResNet to detect PTB, and the model reached an accuracy of 96.73% with a heatmap generation for precise lesion location as well (33). Using 20,135 chest radiographs from 19,681 asymptomatic individuals, an out-of-sample test was conducted (34) to validate the screening performance of the deep learning-based automated detection (DLAD) algorithm developed by Hwang et al. (32). Five images from four active PTB cases confirmed by the bacteriological test were properly classified as having abnormal discoveries with specificities of 0.997 and 0.959 at high specificity and high sensitivity thresholds, respectively. Moreover, DLAD showed a decent performance in identifying radiologically relevant abnormalities with an AUC of 0.967 (95% CI 0.93–0.996). Likewise, to verify the performance of deep learning models on the general population, a study assessed five CNNs in two forms, namely, I-CNN (images input only) and D-CNN [images and demographic variables (age, sex, height, and weight) input] to detect PTB by CXR images in 39,677 workers from Korea. Among the five models, VGG19 achieved the highest performance in both the training and test cohorts, regardless of the demographic information input (AUC 0.9075 of I-CNN and 0.9213 of D-CNN in the test set), and the AUCs of the other four systems were all over 0.88 with D-CNN in the test set. Moreover, no statistical significance was observed when only a single demographic variable was included (*P* > 0.05) (35). Taking advantage of segmentation and augmentation, EfficientNetB3, the CNN structure, demonstrated incredibly high performance in PTB detection with an AUC of 0.999 (36). Moreover, a simplified network was proposed to surmount the trouble of overfitting and difficult deployment in mobile settings owing to the large scale of parameters and hardware requirements of the models, achieving an AUC of 0.925 through 5-fold cross-validation in the diagnosis of PTB on CXRs (37). Uniquely, different from the studies mentioned earlier, Rajaraman et al. blazed new trails to recognize findings consistent with PTB by lateral CXRs through deep learning, with an AUC up to 0.9491 (38).

TB is important not only in the general population but also in patients with specific conditions. Due to the high mortality caused by TB in human immunodeficiency virus (HIV)-positive patients with the conspicuous incidence and improper treatment, in South Africa, Rajpurkar et al. utilized CXRs, as well as certain clinical covariates, including age, temperature, hemoglobin, and white blood cell counts of 677 HIV-positive patients from two hospitals to establish a deep learning algorithm, named CheXaid, which improved the clinicians’ diagnostic accuracy slightly (0.65 vs. 0.60, *P* = 0.002). Interestingly, the performance of the algorithm alone was superior to that of clinicians assisted by AI (accuracy 0.79 vs. 0.65, *P* < 0.001). Moreover, the training strategy of adding clinical variables with CXRs improved the performance of the algorithm (AUC of 0.83 and 0.71 in the combination model and model alone) in this study and suggested the importance of integrating inputs in various modalities to enhance the power of the models (39).

#### Detection of Pulmonary Tuberculosis With Treatment Monitoring and Severity Estimation

Apart from detecting PTB, deep learning is capable to follow post-treatment changes and estimate the severity of it. Utilizing CXRs, the output of the model developed by Lee and his team elevated by 0.30 when the degree of smear positivity increased (*P* < 0.001) and decreased gradually during treatment; meanwhile, the model achieved AUCs over 0.82 in the two test sets for PTB diagnosis (40).

Owing to higher resolution and more subtle presentation, CT images provide more nuanced information on the lung region and play an important role in PTB diagnosis as well (28). Thus, Yan et al. (41) developed a model to detect PTB and quantitatively evaluate the disease burden, of which the quantified TB scores were obviously higher in severe patients than in non-severe ones and was well correlated with the CT scores assessed by radiologists. Moreover, the model demonstrated an accuracy of 83.37% for classifying the six pulmonary lesion types, such as consolidation and calcified granulomas in the validation set, while an accuracy of 98.25% was achieved for distinguishing active PTB patients from inactive individuals in the test set.

The two studies are unique as there is a lack of research targeting treatment monitoring and disease burden estimation of TB by AI methods, which inspires us to launch more relevant studies to give rein to their adjuvant role in the clinic.

#### Validation of Computer-Aided Pulmonary Tuberculosis Detection Systems

In addition to the models obtained from the original studies, the computer-aided detection (CAD) systems, such as qXR, CAD4TB, and Lunit INSIGHT CXR (42), which generate a PTB classification when the output is more than a defined threshold score, have been established to facilitate PTB detection using CXR images based on deep learning. Several studies have exclusively assessed the diagnostic ability of the application of various categories and versions in diverse datasets.

To identify the practicalities of qXR version 2.0 (qXRv2) and CAD4TB version 6.0 (CAD4TBv6) in detecting PTB in low- and middle-income countries with a high disease burden, Khan et al. (43) conducted a prospective single-center study with 2,198 individuals at the Indus Hospital, located in Karachi, Pakistan. Finally, qXRv2 attained a sensitivity of 0.93 (95% CI 0.89–0.95) and a specificity of 0.75 (95% CI 0.73–0.77), while CAD4TBv6 showed a specificity of 0.69 (95% CI 0.67–0.71) when matched with the same sensitivity, both reaching the Target Product Profile recommendations defined by WHO (sensitivity ≥ 0.90 and specificity ≥ 0.70). What’s more, the sensitivity decreased obviously in smear-negative patients compared to that in smear-positive patients (0.80 in the negative group vs. 0.96 in the positive cohort of qXRv2 and 0.82 in the negative population vs. 0.97 in positive individuals of CAD4TBv6). This study is worth emphasizing because it is a rare prospective investigation validating CAD approaches.

Qin et al. (44) estimated three commercially available CAD tools, qXRv2, CAD4TBv6, and Lunit INSIGHT CXR, to triage PTB in 1,196 participants from Nepal and Cameroon, with AUCs above 90% [0.94 (95% CI 0.93–0.96) for Lunit INSIGHT CXR, 0.94 (95% CI 0.92–0.97) for qXRv2, and 0.92 (95% CI 0.90–0.95) for CAD4TBv6]. When the purpose was to reduce the Xpert test by 50%, the sensitivities of the three models maintained at 97–99%, with no statistical significance among them. Subsequently, the group assessed five AI algorithms in newer versions, including CAD4TB version 7 (CAD4TBv7), qXR version 3 (qXRv3), Infereread DR version 2, Lunit INSIGHT CXR version 4.9.0, and JF CXR-1 version 2, on a massive dataset comprising CXRs from 23,954 individuals. The performance of all of them surpassed that of three radiologists as a concrete manifestation that AI showed higher specificity and positive predictive values (PPVs) when matched with the same sensitivity (45). Another study evaluated a maximum of 12 CAD solutions to identify PTB in comparison with an experienced radiologist and an intermediate reader. The final results showed that qXRv3, CAD4TBv7, and Lunit INSIGHT CXR version3.1.0.0 achieved the highest AUC of 0.82. Meanwhile, five of them surpassed the intermediate reader in specificity and accuracy when holding at the same sensitivity, while only qXRv3 maintained comparable specificity when sensitivity reached the standard of the experienced reader [95.5% (95% CI 90.4–98.3%)] (46). The three studies mentioned (43, 45, 46) coincidentally discovered that, in groups with previous TB, the performance of AI systems would decline to some extent. In addition, when integrating clinical information with the CAD scores of CXRs generated by CAD4TB, the AUC of the combination framework reached 0.84, improving the performance of CAD4TB alone (47).

Although the verification results seem remarkable as a whole, more prospective validation tests need to be carried out in a real medical environment, after which these mercantile AI systems may be competent enough to supply convenient, efficient, and accurate tools for physicians worldwide, facilitating clinical decision making in the near future.

### Discrimination Between Pulmonary Tuberculosis and Other Lung Diseases

In addition to detection, effort has been made to differentiate PTB from other pulmonary diseases (Table 3).

**TABLE 3:** Summary of AI applications in discrimination between pulmonary tuberculosis and other lung diseases.

No.	References	Method	Reference standard	Dataset	Study population	Discrimination	Training/Validation/test cohort	Model names	Algorithm	Results
1	Feng et al. (52)	Retrospective multi-center on CT images	Histological diagnosis	550 patients	China	PTB and lung cancer	Training:218 Internal validation:140 External validation: 192	NA	DLN	AUC 0.809, Sen 0.908, Spe 0.608, Acc 0.828 in the external validation set
2	Zhuo et al. (53)	Retrospective multi-center on CT images	Surgical pathology, specimen culture or assay	313 patients	China	PTB and lung cancer	Training: validation 7:3	NA	Radiomics nomogram	AUC 0.99, Sen 0.9841, Spe 0.9000, Acc 0.9570 in the validation set
3	Hu et al. (54)	Retrospective multi-center on PET/CT images	Pathological or follow-up confirmation	235 patients	China	PTB and lung cancer	Training: 163 Validation: 72	NA	Radiomics nomogram	AUC 0.889, Sen 85%, Spe 78.12%, Acc 79.53% in the validation set
4	Du et al. (55)	Retrospective single-center on PET/CT images	Pathology	174 patients	China	PTB and lung cancer	Training: 122 Validation: 52	NA	Radiomics nomogram	AUC 0.93, Sen 0.86, Spe 0.83, Acc 0.85 in the validation set
5	Wang et al. (59)	Retrospective multi-center on CT images	Sputum acid-fast bacilli stain or culture	1,185 patients	China	MTB-LD and NTM-LD	Training: validation: test 8:1:1 External test: 80	NA	CNN	AUC 0.78, Sen 0.75, Spe 0.63, Acc 0.69 in the external test set
6	Yan et al. (60)	Retrospective multi-center on CT images	Sputum culture or smear	182 patients	China	MTB-LD and NTM-LD	Training: validation 8:2 External validation: 40	NA	Radiomics	AUC 0.84—0.98, Sen 0.61–0.97, Spe 0.61–0.97 in the external validation set

*AI, artificial intelligence; CT, computed tomography; PTB, pulmonary tuberculosis; NA, not available; DLN, deep learning nomogram; AUC, area under the curve; Sen, sensitivity; Spe, specificity; Acc, accuracy; PET/CT: positron emission tomography/computed tomography; MTB-LD, Mycobacterium tuberculosis lung disease; NTM-LD, non-tuberculous mycobacterium lung disease; CNN, convolutional neural network.*

#### Discrimination Between Tuberculosis and Lung Cancer

Lung cancer is one of the primary causes of cancer death and is the most common tumor worldwide (48). Moreover, pulmonary tuberculosis granuloma (TBG) may present as lung adenocarcinoma (LAC) with the demonstration of similar solitary pulmonary nodules (49–51), resulting in diagnostic confusion and treatment mistakes. A deep learning-based nomogram (DLN) using CT images was developed and validated to distinguish TBG from LAC (52). The DLN was constituted to compare with a clinical model including age, sex, and subjective findings on CT images, and a deep learning signature (DLS) model, with scores derived from 14 deep learning features constructed in advance, and showed better diagnostic performance than the clinical and DLS models. Comprised by age, sex, lobulated shape, and DLS score, DLN achieved both higher AUC and sensitivity than the other 2 models in the internal validation cohort, meanwhile showing an AUC of 0.809 in the external validation set. A radiomics nomogram based on CT images was proposed by another group, showing an AUC of 0.99 in the validation set to differentiate the two fundamentally different diseases which demonstrated similarities between each other (53). Analogously, to distinguish between solitary LAC and PTB, Hu et al. constructed a radiomic model containing a set of nine fluorine-18-fluorodeoxyglucose PET/CT (18F-FDG PET/CT) radiomic features, such as Histogram_Skewness and SHAPE_Sphericity (54). While developing a clinical model, they also constructed a complex model, which was a combination of the radiomic and clinical models using multivariate LR. Finally, the radiomic and complex models outperformed the clinical model, as the AUC of the complex model reached 0.909, while the radiomic and clinical models achieved 0.889 and 0.644 in the validation set. Furthermore, a similar study utilized a radiomic nomogram integrating the radiomic score (RAD-score) derived from a weighted linear combination of features selected from 18F-FDG PET/CT images and three semantic features to differentiate the two semblable image phenotypes. The diagnostic performance of the radiomic nomogram slightly surpassed that of the radiomic and semantic models with an AUC of 0.93 in the validation cohort; the decision curve also illustrated the net benefit of the nomogram (55).

#### Discrimination Between Tuberculosis and Non-tuberculous Mycobacterium Lung Disease

Given that non-tuberculous mycobacterium lung disease (NTM-LD) demonstrates an increasing incidence and prevalence in recent years (56, 57), due to similar clinical symptoms and CT imaging characteristics with mycobacterium pulmonary tuberculosis lung disease (MTB-LD) (58), it is crucial to distinguish the different infections as quickly as possible in the early stage to permit appropriate treatment implementation. A deep learning framework was developed by Wang and his colleagues to differentiate between NTM-LD and MTB-LD on chest CT images with an AUC of 0.86 and 0.78 in the internal test set and in the external test cohort, respectively (59). Moreover, the model surpassed three radiologists in almost every metric with higher diagnostic efficiency (1,000 times faster) and output class activation maps identifying abnormal lung areas without manual annotation. To achieve a similar purpose, another study leveraged radiomics by taking advantage of the features of cavities in CT images using six machine learning models (SVM, RF, LR, etc.) (60); 458 ROIs were depicted by two radiologists, with 29 optimal quantified image features, such as gradient and wavelet, selected subsequently. AUCs of the six models were up to over 0.98 in the training and validation sets.

These studies pioneered the application of AI for the discrimination of PTB from lung cancer and NTM-LD, with promising results encouraging investigators to develop more AI models using a variety of original training materials to differentiate PTB from more diseases.

### Identification of Tuberculosis Drug Resistance

In the context of increasing incidence and intractable management of TB resistance, multiple examination approaches, including drug susceptibility testing (DST), Xpert MTB/RIF, line-probe assays, and whole-genome sequencing (WGS), have been explored to identify DR-TB (2). However, cost and time issues are still remaining. Hence, inexpensive, rapid, and accurate tools for automated detection of the antimicrobial resistance are of great concern (Table 4).

**TABLE 4:** Summary of AI applications in TB drug resistance identification.

No.	References	Method	Reference standard	Dataset	Study sample	Resistance identification	Training/Validation/test cohort	Model names	Algorithm	Results
1	Jaeger et al. (63)	Retrospective multi-center on CXR images	NA	135 patients	Belarus	MDR-TB	5-fold cross validation	NA	ANN, CNN and ML	AUC 50–66%, Acc 0.62–0.66
2	Karki et al. (64)	Retrospective multi-center on CXR images	DST	5,642 CXR images	United States, China, etc.	DR-TB	10-fold cross validation	NA	CNN	AUC 0.85
3	Gao and Qian (65)	Retrospective multi-center on CT images	NA	230 patients	NA	MDR-TB	Training: 150 Validation: 35 Test: 45	NA	CNN and ML	Acc 64.71–91.11%
4	Yang et al. (68)	Retrospective multi-center on gene sequences	DST	8,388 isolates	European, Asia, and Africa	4 drugs and MDR-TB	Training: test 7:3	DeepAMR	ML	AUC 94.4–98.7%, Sen 87.3–96.3%, Spe 90.9–96.7%
5	Yang et al. (69)	Retrospective multi-center on gene sequences	DST	13,402 isolates	NA	4 drugs	Training: validation: test 4:2:2 or stratified cross validation	HGAT-AMR	GNN	AUC 72.83–99.10%, Sen 50.65–96.60%, Spe 79.50–98.87%
6	Yang et al. (70)	Retrospective multi-center on gene sequences	DST	1,839 isolates	United Kingdom	8 drugs and MDR-TB	Cross-validation	NA	ML	AUC 91–100%, Sen 84–97%, Spe 90–98%
7	Deelder et al. (71)	Retrospective multi-center on gene sequences	DST	16,688 isolates	NA	14 drugs and MDR-TB	5-fold cross validation	NA	ML	Acc 73.4–97.5%, Sen 0–92.8%, Spe 75.6–100%
8	Chen et al. (72)	Retrospective multi-center on gene sequences	DST	4,393 isolates	ReSeqTB Knowledgebase	10 drugs	10-fold cross validation Independent validation: 792	NA	WDNN and ML	AUC 0.937, Sen 87.9%, Spe 92.7% for the first-line drugs
9	Gröschel et al. (73)	Retrospective multi-center on gene sequences	DST	20,408 isolates	NCBI Nucleotide Database	10 drugs	Training: validation 3:1	GenTB	WDNN and ML	AUC 0.73–0.96, Sen 57–93%, Spe 78–100%
10	Kuang et al. (75)	Retrospective multi-center on gene sequences	DST	10,575 isolates	China, Cameroon, Uganda, etc.	8 drugs	10-fold cross validation	NA	CNN and ML	Acc 89.2–99.2%, Sen 93.4–100%, Spe 48.0–91.7%, F1 score 93.3–99.6%
11	Jiang et al. (76)	Retrospective multi-center on gene sequences	DST	12,378 isolates	NCBI-SRA Database	4 drugs	Training: validation: test 8:1:1 and 10-fold cross validation	HANN	Attentive neural network	AUC 93.66–99.05%, Sen 67.12–96.31%, Spe 92.52–98.84%

*AI, artificial intelligence; TB, tuberculosis; CXR, chest X-ray; NA, not available; MDR-TB, multi-drug resistant tuberculosis; ANN, artificial neural network; CNN, convolutional neural network; ML, machine learning; AUC, area under the curve; Acc, accuracy; DST, drug susceptibility testing; DR-TB, drug-resistant tuberculosis; CT, computed tomography; Sen, sensitivity; Spe, specificity; GNN, graph neural network; WDNN, wide and deep neural network; SRA, sequence read archive.*

#### Drug-Resistant Tuberculosis Identification Based on Medical Images

Imaging manifestations of these two main categories of TB, sensitive or resistant to anti-tuberculosis therapy (ATT), differ depending on the phenotypes, as DR-TB could demonstrate larger lesions and thick-walled cavities on CXR images (61, 62). Jaeger et al. (63) trained an artificial neural network through cross-validation to identify patients with multi-drug resistant tuberculosis (MDR-TB) using CXRs, which achieved an AUC of only up to 66%. This unsatisfactory result may be explained by the small dataset containing only 135 cases. However, it is inspiring that the team used a larger dataset of 5,642 CXRs and various CNNs for the same purpose, and finally, a preferable outcome was obtained. With static or dynamic data augmentation, the AUC of InceptionV3 increased to 0.85. For custom CNNs, six-layer CNN expressed the best performance with an AUC of 0.74 (64). After the ImageCLEF2017 competition, a study utilized a small dataset from the match, which comprised CT images from 230 drug-sensitive and MDR-TB patients to implement a combination of a patch-based deep CNN and SVM, with an accuracy of 91.11% in predicting MDR-TB at the patient level and 79.8% at the patch level (65).

To date, the exploitation of using medical images to identify DR-TB has not been investigated thoroughly; hence, these studies are noteworthy because they could give us some instructions for future research orientation.

#### Drug-Resistant Tuberculosis Identification Based on Genetic Data

Besides medical images, genetic information could also serve as a diagnostic tool for TB. As introduced above, various molecular approaches are capable of detecting drug resistance, of which the theoretical proof is that the resistance occurrence in TB is caused by chromosomal mutations, passing along through vertical descent, in present genes. Meanwhile, rapid molecular tests using genomic information are more efficient than culture-based assays so they are adopted widely, and related gene data are available for scientific research (66). Therefore, numerous AI studies based on gene sequences have been explored to identify drug resistance of *M. tuberculosis*, as follows.

As researched previously, deep learning using genomic data has been applied to reveal antibiotic resistance (67). Thus, with mutations for isolates input and phenotypes of drug resistance output, Yang et al. (68) developed “DeepAMR,” a deep learning model with a deep denoising auto-encoder for multiple tasks to predict co-occurrent drug resistance of *M. tuberculosis*, comparing the model with conventional machine learning methods, including RF, SVM, and ensemble classification chains (ECC). The co-occurrence of rifampicin (RIF) and isoniazid (INH) resistance accounted for the majority of the dataset (*n* = 8,388). The results suggested that the model surpassed all other approaches in predicting resistance to the four first-line drugs, MDR-TB, and pan-susceptible tuberculosis (PANS-TB, isolates susceptible to any of the four first-line drugs), showing AUCs from 94.4 to 98.7% (*P* < 0.05). Later, utilizing a novel method using graphs translated from genetic data of *M. tuberculosis*, the team developed a graph neural network named “HGAT-AMR” to predict drug resistance in a sample consisting of 13,402 isolates tested for susceptibility to up to 11 drugs (69). HGAT–AMRi-E (HGAT–AMR trained on any available incomplete phenotype specimen for the multi-label learning task) and HGAT–AMRs (HGAT–AMR trained on individual subsets of different drugs for the single-label learning task) performed best in INH and RIF, respectively, with AUCs of 98.53 and 99.10%. Meanwhile, HGAT–AMRi-E demonstrated the highest sensitivity for INH, ethambutol (EMB), and pyrazinamide (PZA) at 94.91, 96.60, and 90.63%, respectively, and HGAT-AMR outperformed SVM and LR, unless in a condition of highly imbalanced data when an isolate had only been tested by INH and EMB, but not by other drugs. Favorable performance was yielded in machine learning models constructed by the group as well, with higher sensitivity compared to the previous rule-based method (*P* < 0.01) (70). Collecting 16,688 isolates of which the WGS and DST data are available to predict drug resistance, another study developed the gradient boosted tree, a machine learning method, reaching an accuracy of 95.5% in MDR-TB identification (71).

Similarly, to determine the drug resistance of *M. tuberculosis* strains by inputting gene sequences, Chen et al. compared the performance of three deep learning models (72). The wide and deep neural network (WDNN), constructed in the study, incorporating LR and deep multilayer perceptron, was presented in four forms, namely, kSD-WDNN (detecting preselected mutations), SD-WDNN (detecting single resistance), and 2 MD-WDNNs (detecting common mutations and for all mutations in multiple resistance), in which the most complex model MD-WDNN surpassed others in both first-line and second-line drugs, with average AUCs of 0.937 and 0.891 in the validation set. Subsequently, a correlative study developed a user-friendly online tool named GenTB based on genome sequencing to predict the antibiotic resistance (73), involving the WDNN and an RF algorithm constituted by Farhat et al. (74). After testing on 20,408 isolates, both GenTB-RF and GenTB-WDNN demonstrated satisfactory performance in first-line drugs with AUCs of more than 87% and with a slightly lower performance in second-line drugs. In particular, GenTB-RF reached the highest prediction for RIF [AUC 96% (95% CI 95–96%)]. Based on 1D CNN, using large and diverse *M. tuberculosis* isolates from six continents to verify the accuracy and steadiness of deep learning, another study developed a model which outperformed the advanced Mykrobe classifier which utilizes a De Bruijn graph to identify resistance profiles in antimicrobial-resistant prediction with higher F1 scores (75). Concurrently, it is worth mentioning that an innovative hierarchical attentive neural network has been constructed to predict the drug resistance of *M. tuberculosis* through genome-wide variants recently, discovering a potential gene related to drug resistance besides achieving supernal AUC and sensitivity in resistance recognition (76).

## Discussion

As described earlier, in terms of TB detection, discrimination, and drug resistance identification, AI showed a great potential, with performance approximate to or even better than that of physicians. Yet, there are still lots of challenges remaining, with the concurrence of prospects, as described below.

### Challenges

First, DR-TB remains a critical issue worldwide, with an increasing incidence and tough management. Developing dependable AI systems using sufficient radiology-based data, which is more convenient than gene sequences to rapidly recognize patients with DR-TB to assist physicians in executing correct clinical decisions, is of great imperative.

Then, up till now, only a few studies have adopted deep learning or other AI approaches to predict TB relapse or treatment response to anti-tuberculosis drugs. An algorithm based on CNN was proposed to predict the persistence time needed to achieve culture negative in TB individuals with an unsatisfactory accuracy, regrettably (77). In addition, it has been revealed that the minimum inhibitory concentration grew higher with an increasing risk of relapse (78) and aggressive regimens may reduce the recurrence of MDR-TB after successful treatment (79). Thus, if a prediction of relapse can be made in advance, more precise and positive treatment could be carried out to reduce the hazard of returning.

Third, the generalization of these models in a broader population remains to be seen, since not all of those studies contain external tests, and research samples are not abundant or variable enough. However, studies, including external validation sets, demonstrated diminishing performance from training to external cohorts which gives us a hint of sustaining the reproducibility of the models to suit various individuals. Perhaps, multicenter studies in an enormous study population are capable of solving this problem, but the subsequent issues of data transmission efficiency and security in the process of data sharing deserve to be highlighted.

As for model modalities, since Lu et al. developed a fusion CNN integrated with images and basic clinical information to predict lung cancer (80) and the model CheXaid utilized CXRs with clinical variables to detect TB in HIV patients (39), it is being probed prevalently in the construction of a fusion neural network, which is composed of several modules dealing with data at multiple scales. Thus, there is an incredible amount of untapped potential to develop AI models with the capacity to handle multimodal inputs. Furthermore, primary inputs, including images or data in other forms, are supposed to be standardized, while diversiform data obtained from different apparatuses may be at an uneven quality level.

Finally, to achieve the purpose of directing clinical practice, the practicality of these novel models should be tested in a real medical environment and seamlessly integrated into the routine workflow, especially in countries with high TB burden and a lack of advanced medical equipment and professional physicians. Owing to the prospective real-world clinical setting, the superior performance of retrospectively developed AI compared with that of human should be regarded with some care.

### Prospect

Following tremendous progress in computational power and advanced techniques, AI is blooming increasingly in countless fields. In radiology, AI demonstrates remarkable performance in the detection, treatment monitoring, and prognosis prediction of multiple diseases, especially in oncology. With regard to TB, saving labor and time costs, AI is capable of improving detection efficiency and precision; therefore, medical institutions worldwide could benefit from these novel assistance tools. In the coming decades, after better integration with clinical workflow, AI will exert a brilliant influence on the entire duration of TB from screening, diagnosis, and treatment following to outcome prediction, meanwhile saving medical resources, avoiding inappropriate management, and improving the quality of life of patients.

## Conclusion

AI-based approaches, including deep learning, radiomics, and other conventional machine learning methods applied to TB, provide a self-driven, convenient, and time-saving strategy to improve diagnostic efficiency and accuracy, outperforming radiologists. Nonetheless, the clinical utility of them remains to be verified, while pitfalls, such as reproducibility of the model and data standardization, need to be addressed as well. To summarize, in this review, we listed several studies focusing on AI-based assistance methods applied to TB detection, discrimination, and drug resistance identification using CXR, CT, PET/CT images, and genome data. Although most of these studies developed AI models with favorable performance, quite a few hurdles must be overcome along the way to maximize the potential of AI. Although TB is especially emphasized in this study, application of AI in other diseases is worth equivalent attention.

## Author Contributions

WL and CW contributed to supervision and conceptualization. SL, GW, JS, and JL designed the search strategy, performed the literature retrieval, and contributed to the original draft of the manuscript. CW, HD, JM, and SL reviewed and revised the final version of the manuscript. WL contributed to the funding acquisition. All authors read and approved the submitted version.

## Conflict of Interest

The authors declare that the research was conducted in the absence of any commercial or financial relationships that could be construed as a potential conflict of interest.

## Publisher’s Note

All claims expressed in this article are solely those of the authors and do not necessarily represent those of their affiliated organizations, or those of the publisher, the editors and the reviewers. Any product that may be evaluated in this article, or claim that may be made by its manufacturer, is not guaranteed or endorsed by the publisher.
