# Supplementary material for: The Application of Artificial Intelligence in the Diagnosis and Drug Resistance Prediction of Pulmonary Tuberculosis
Source: Front Med (Lausanne). 2022 Jul 28;9:935080. doi: 10.3389/fmed.2022.935080 (PMC9366014; doi:10.3389/fmed.2022.935080)
Supplement: Supplementary file 1 [file Data_Sheet_1.DOCX]

Supplementary Material

## Supplementary Figures


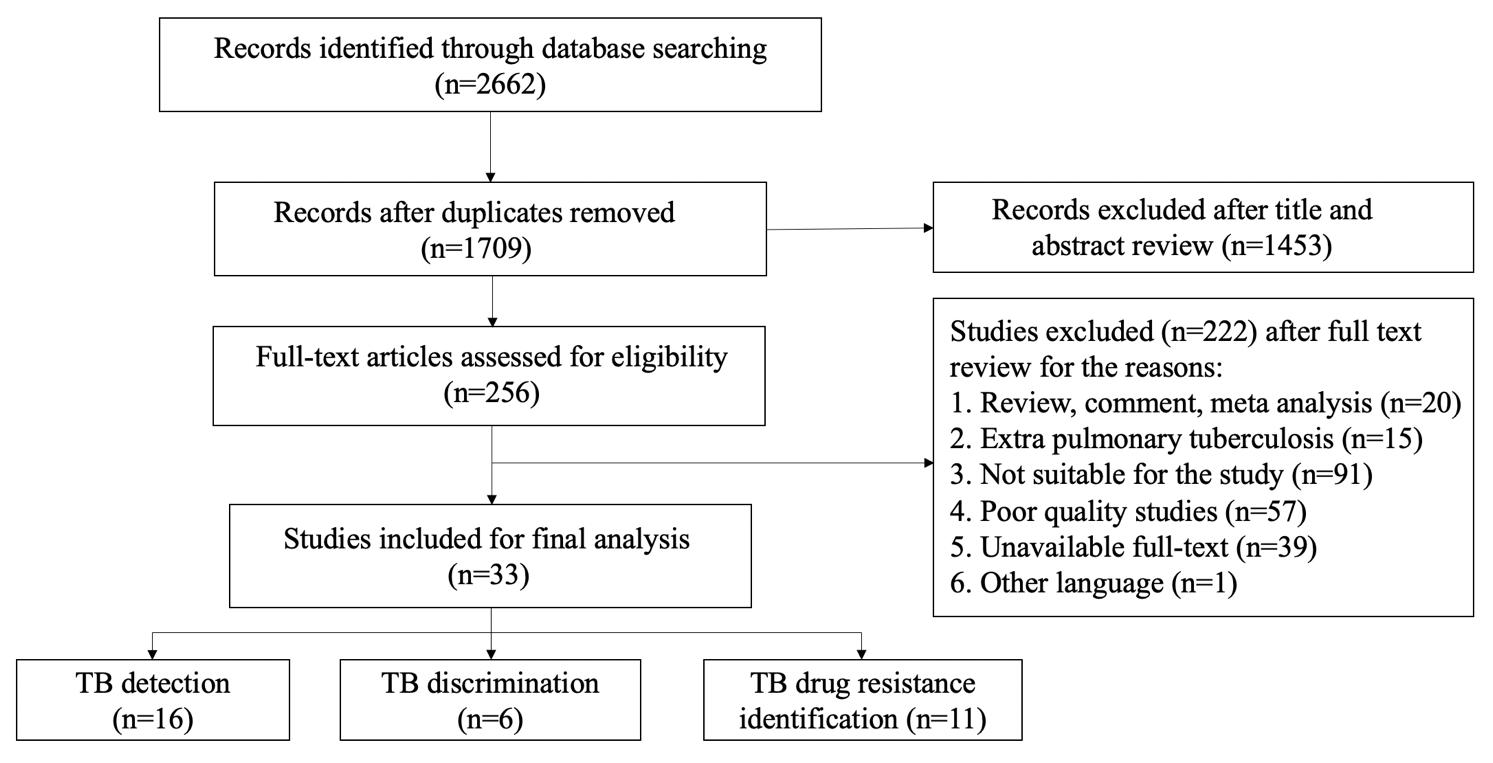


**Supplementary Figure 1.** Flowchart for literature search and selection.
